# Supplementary material for: Efficacy and safety of saroglitazar for the management of dyslipidemia: A systematic review and meta-analysis of interventional studies
Source: PLoS One. 2022 Jul 1;17(7):e0269531. doi: 10.1371/journal.pone.0269531 (PMC9249226; doi:10.1371/journal.pone.0269531)
Supplement: S1 Table — (DOCX) [file pone.0269531.s002.docx]

| **Database** | **Search Strategy** |
| --- | --- |
| PubMed | ((("alpha-ethoxy-4-(2-(2-methyl-5-(4-methylthio)phenyl))-1H-pyrrol-1-yl)ethoxy))benzenepropanoic acid") OR (saroglitazar)) OR ("ZYH1 compound")) AND (((hyperlipidemia) OR (dyslipidemia)) OR (hypertriglyceridemia)) |
| Scopus | ALL ( saroglitazar ) OR ALL ( "alpha-ethoxy-4-(2-(2-methyl-5-(4-methylthio)phenyl))-1H-pyrrol-1-yl)ethoxy))benzenepropanoic acid" ) OR ALL ( "ZYH1 compound" ) |
| Cochrane | ("alpha-ethoxy-4-2-2-methyl-5-4-methylthiophenyl-1H-pyrrol-1-ylethoxy benzenepropanoic acid" OR saroglitazar OR "ZYH1 compound") AND (hyperlipidemia OR dyslipidemia OR hypertriglyceridemia) |

**Appendix 1:** Search Strategy
