# Supplementary material for: Efficacy and safety of saroglitazar for the management of dyslipidemia: A systematic review and meta-analysis of interventional studies
Source: PLoS One. 2022 Jul 1;17(7):e0269531. doi: 10.1371/journal.pone.0269531 (PMC9249226; doi:10.1371/journal.pone.0269531)
Supplement: S2 File — (DOCX) [file pone.0269531.s004.docx]

**Supplementary Table 1:** Quality assessment of single arm studies

| Study | **1** | **2** | **3** | **4** | **5** | **6** | **7** | **8** | **9** | **10** | **11** | **12** | **Quality Rating** |
| --- | --- | --- | --- | --- | --- | --- | --- | --- | --- | --- | --- | --- | --- |
| Bhosle D et al., 2018 | Yes | Yes | Yes | Yes | Yes | Yes | Yes | No | Yes | Yes | Yes | No | **Good** |
| Deshpande A et al., 2016 | Yes | Yes | Yes | Yes | Yes | Yes | Yes | No | Yes | Yes | Yes | No | **Good** |

1: Study question; 2: Eligibility criteria and study population; 3: Study participants representative of clinical populations of interest; 4: All eligible participants enrolled; 5: Sample size; 6: Intervention clearly described; 7: Outcome measures clearly described, valid, and reliable; 8: Blinding of outcome assessors; 9: Followup rate; 10: Statistical analysis; 11: Multiple outcome measures; 12: Group-level interventions and individual-level outcome efforts
