# Supplementary material for: Efficacy and safety of saroglitazar for the management of dyslipidemia: A systematic review and meta-analysis of interventional studies
Source: PLoS One. 2022 Jul 1;17(7):e0269531. doi: 10.1371/journal.pone.0269531 (PMC9249226; doi:10.1371/journal.pone.0269531)
Supplement: Attachment — Submitted filename: Rebuttal letter_R2.docx [file pone.0269531.s006.docx]

**REBUTTAL LETTER-R2**

Respected Editor-In-Chief

Thank you for your timely review of our manuscript entitled “Efficacy and safety of Saroglitazar for the management of Dyslipidemia: A systematic review and meta-analysis of interventional studies” [PONE-D-21-19447]. We have gone through all of your valuable comments. Please find the revisions and justifications made based on comment to comment in this rebuttal letter and the same has been updated in the revised manuscript (R2) with track changes.

**Reviewer #1:** Dr Chhabra and colleagues have conducted a systematic review of interventional studies for saroglitazar and included 4 studies in their meta-analysis. I appreciate the authors efforts to respond to the first round of comments and I can see they have made significant revisions to strengthen their manuscript. However, I still have several concerns regarding the methods and presentation of the results. My major concern is about the latest date of the literature search which was performed 22 months ago (May 2020).

**Comment 1#**

Have the authors updated their search of the literature? The date of the last search was May 2020 which is not very recent. I will like to see an updated literature search in case recently published studies have been missed.

**Response#**

Yes, we have updated the literature search in January 2022 and we could identify a single new study related to Saroglitazar, unfortunately, that study didn’t meet our inclusion criteria.

We have updated the same in our methodology part which is as follow

“The initial search was performed in April 2019 and was updated in January 2022.”

**Comment 2#**

Abstract, results: Total cholesterol and LDL cholesterol, saraglitazar 4 mg vs control does not match the number of studies included in the results in the text and (Figures 5 and 7 only have 3 studies).

The sentence (line 67) to the abstract “Saroglitazar showed a reduction in liver transaminases and serum creatinine” is too vague. I suggest you add the same details as the earlier sentences (intervention, comparator, estimate, p value # of studies).

**Response#**

Thanks for the picky point we have rectified number of studies from 4 to 3

“and control [SMD: −0.36 mmol/L, 95% CI −0.59 to -0.12; p < 0.01; 3 studies].”

“[SMD − 0.49 mmol/L, 95% CI: − 0.72 to -0.26; p < 0.0001; 3 studies].”

Considering you suggestion about the vague sentence we have removed it from the abstract and instead of the there was requirement of the sentence, we kept new sentence as “Saroglitazar was not associated with any serious adverse events.”

**Comment 3.** P values for estimates should not be dichotomized at 0.05 level. Please include the actual p value for the pooled estimates in the abstract and results.

**Response#**

Thanks for your suggestion. As you suggested, we have replaced dichotomized P-values to exact P-Values in both abstract and the results section.

**Comment 4.** Please add the unit of measurement for lipids (mmol/L) be added to the forest plot figure titles and Table 3.

**Response#**

Thanks for your suggestion. As you suggested, we have added the unit of measurement in the forest plot figure titles, Table 3, abstract, and the text in the results section for each and every included outcome

**Comment 5**. There is a discrepancy between figure 1 tiff file and figure 1 docx file in the number of studies included in meta-analysis.

**Response#**

This was happened as we updated Figure 1 during revision and we accidently forgot remove the tiff file submitted during the initial submission. Now we have attached the final figure 1 (R2) and removed the old file from the submission

**Comment 6**

Language issues:

Abbreviations: Sometimes LDL cholesterol and LDL-cholesterol (recommend not using the dash as this seems to be the norm for most publications).

**Response#**

Rectified as per your suggestion, Thanks

**Comment 7**

Line 2, line 51 and others: No need to specifically capitalize saroglitazar and dyslipidemia

**Response#**

We have considered your suggestion and changes made are in whole context of the manuscript can be seen as track on mode

**Comment 8**

Line 431: use and not &

**Response#**

Rectified, thanks for your suggestion

**Comment 9**

Line 435: misspelled trials as trails.

**Response#**

Rectified, thanks for your suggestion

**Comment 10**

Throughout the paper the authors should consistently use the term dyslipidemia which, to my understanding, encompasses any problematic concentration of blood lipid measures such as hyperlipidemia, hypertriglyceridemia or low HDL cholesterol.

**Response#**

Thanks for the suggestion, we have rectified it

**Reviewer #2:**

Thank you for the revision. The majority of concerns have been addressed. Before the manuscript is acceptable for publication, I think a few issues need to be revised again.

**Comment 1#**

First, some more useful information should be added to the abstract section. For the method, it is not necessary to state “Two individual reviewers performed an independent review for study selection, data extraction and quality assessment.”. A summary description of the selection of outcomes or the method of data synthesis can be provided. And for the results part, it is not comprehensive to describe only the significant results. All study results should be summarized because you have discussed them in the discussion section.

**Response#**

We have considered your suggestion and we have added the outcomes considered and details on quality assessment in the methodology part of the abstract which is as follows:

“The efficacy of saroglitazar was assessed with respect to its effect in total cholesterol, LDL and HDL-cholesterol, triglycerides, fasting plasma glucose, and non-HDL cholesterol. The effects on serum creatinine levels, bodyweight reduction, alanine aminotransferase and aspartate aminotransferase were considered to be safety endpoint. The Cochrane risk of bias assessment tool was used to assess the methodological quality of the included studies”

**Comment 2#**

Secondly, I agree with reviewer 1 that avoiding using the terms statistically significant or non-significant decrease (not only for TG). Except for TC and LDL-C, the P-values for other results are greater than 0.05, we do not know if there was a truly decrease. And in the discussion and conclusion part, “the overall results of this study are encouraging with a reduction in fasting plasma glucose, triglycerides, total cholesterol, LDL-cholesterol,” such concluding statement exaggerates your results, the significance and strength of the evidence do not support such a conclusion. Perhaps you can say that such results (TG, FPG) could be considered suggestive.

**Response#**

We have modified such exaggerating results as per the reviewer’s comments

**Comment 3#**

Thirdly, I don’t agree the statement that Saroglitazar showed a reduction in liver transaminases, serum creatinine and body weight (abstract line 67 and discussion line 379), as the results for Saroglitazar 4mg vs control were not significant and the results for 4mg vs control and 4mg vs 2mg were reversed for body weight and Aspartate Amino transferase. It can only be said that there is no evidence that Saroglitazar has effects on these safety outcomes. And the SMD for serum creatinine were positive, which is inconsistent with what is stated in the abstract and discussion.

**Response#**

We have carefully gone through the statement and have removed the sentence from the abstract, Thanks for your kind suggestion.

7. PLOS authors have the option to publish the peer review history of their article ([what does this mean?](https://journals.plos.org/plosone/s/editorial-and-peer-review-process#loc-peer-review-history)). If published, this will include your full peer review and any attached files.


If you choose “no”, your identity will remain anonymous but your review may still be made public.


**Do you want your identity to be public for this peer review?** For information about this choice, including consent withdrawal, please see our [Privacy Policy](https://www.plos.org/privacy-policy).

Reviewer #1: **Yes:** Joseph Blais

Reviewer #2: **Yes:** Xin Huang
