# Supplementary material for: Efficacy and safety of saroglitazar for the management of dyslipidemia: A systematic review and meta-analysis of interventional studies
Source: PLoS One. 2022 Jul 1;17(7):e0269531. doi: 10.1371/journal.pone.0269531 (PMC9249226; doi:10.1371/journal.pone.0269531)
Supplement: Attachment — Submitted filename: Rebuttal letter_R3.docx [file pone.0269531.s007.docx]

**REBUTTAL LETTER-R4**

Respected Editor-In-Chief

Thank you for your timely review of our manuscript entitled “Efficacy and safety of Saroglitazar for the management of Dyslipidemia: A systematic review and meta-analysis of interventional studies” [PONE-D-21-19447]. We have gone through all of your valuable comments. Please find the revisions and justifications made based on comment to comment in this rebuttal letter and the same has been updated in the revised manuscript (R3) with track changes.

**Comment 1**

Please ensure that you refer to Table 1 in your text as, if accepted, production will need this reference to link the reader to the Table.

**Response#**

Based on the reviewer comment, we referred Table 1 in the text before the appearance of table.

**Comment 2**

Please include your tables as part of your main manuscript and remove the individual files.

**Response#**

Based on the reviewer comment, we included tables as part of the manuscript and removed individual files.

***Thanks for reviewing our paper and providing us with the comments to improve further***
